# Higher Tactile Temporal Resolution as a Basis of Hypersensitivity in Individuals with Autism Spectrum Disorder

**DOI:** 10.1007/s10803-018-3677-8

**Published:** 2018-07-17

**Authors:** Masakazu Ide, Ayako Yaguchi, Misako Sano, Reiko Fukatsu, Makoto Wada

**Affiliations:** 1 Developmental Disorders Section, Department of Rehabilitation for Brain Functions, Research Institute of National Rehabilitation Center for Persons with Disabilities, 4-1, Namiki, Tokorozawa-shi, Saitama, 359-8555 Japan; 2 Japan Society for the Promotion of Science, Tokyo, Japan; 3 Department of Contemporary Psychology, Rikkyo University, Saitama, Japan; 4 Information and Support Center for Persons with Developmental Disorders, National Rehabilitation Center for Persons with Disabilities, Saitama, Japan; 5 National Rehabilitation Center for Children with Disabilities, Tokyo, Japan; 6 Department of Rehabilitation for Brain Functions, Research Institute of National Rehabilitation Center for Persons with Disabilities, Saitama, Japan

**Keywords:** Hypersensitivity, Autism spectrum disorder, Temporal order judgment, Detection threshold/sensitivity, E/I imbalance, Tactile

## Abstract

Many individuals with autism spectrum disorder (ASD) have symptoms of sensory hypersensitivity. Several studies have shown high individual variations in temporal processing of tactile stimuli. We hypothesized that these individual differences are linked to differences in hyper-reactivity among individuals with ASD. Participants performed two tasks as to vibrotactile stimuli: One is a temporal order judgement task, and another is a detection task. We found that individuals with ASD with higher temporal resolution tended to have more severe hypersensitivity symptoms. In contrast, the tactile detection threshold/sensitivity were related to the severities of stereotyped behaviour and restricted interests, rather than to hypersensitivity. Our findings demonstrate that higher temporal resolution to sensory stimuli may contribute to sensory hypersensitivity in individuals with ASD.

**Electronic supplementary material:**

The online version of this article (10.1007/s10803-018-3677-8) contains supplementary material, which is available to authorized users.

## Introduction

Individuals with autism spectrum disorder (ASD) not only show deficits in social communication, but also show atypical sensory processing characterized by sensory hypersensitivity. This feature is also emphasized in the fifth edition of the Diagnostic and Statistical Manual of Mental Disorders (DSM-5) (APA 2013). Sensory dysfunction such as this can be assessed using the Sensory Profile, which is a self-report questionnaire that determines the individual’s sensitivity; for example, if he/she shows strong emotional responses to sensory stimuli experienced as a part of daily life (Dunn 1999; Dunn and Westman 1997). Tomchek and Dunn (2007) reported that children with ASD are more sensitive to sensory information (tactile, 65.1%; taste and olfaction, 56.2%; visual and auditory, 50.9%) than typically developing (TD) peers (13, 9.7, and 7.9%, respectively) according to their scores on a short version of the Sensory Profile (i.e. Short Sensory Profile).

Sensory hypersensitivity is conventionally explained by the hypothesis that an abnormally high sensitivity for detecting sensory signals leads to atypical responsiveness. Blakemore et al. (2006) reported that individuals with Asperger’s syndrome can detect small displacements in vibrotactile stimuli with a lower detection threshold than TD control individuals; this feature remained dominant even when the stimuli were delivered at a high frequency (200 Hz). Cascio et al. (2008) and Puts et al. (2014) demonstrated that the lower detection thresholds were found even when the vibrotactile stimuli were presented at low frequencies (33 and 25 Hz). However, another study showed that the detection thresholds of children with ASD and TD children were not significantly different regardless of stimulus frequency (40 and 250 Hz) (Guclu et al. 2007). These contrasting results from previous studies may partly be due to the inherent variability in sensory processing of individuals with ASD (Simon and Wallace 2016). Thus, individual differences in sensory processing must be accounted for and it should be considered how they relate to varied responsiveness to sensory stimuli in patients’ with ASD daily life.

Another hypothesis that can explain hypersensitivity in patients with ASD involves aberrant temporal processing of sensory inputs. Individuals with ASD frequently complain about the flickering nature of fluorescent illumination, which is also thought to induce their repetitive behaviour (Colman et al. 1976). These findings might indicate that some patients with ASD might have extremely high temporal resolution (exceeding the 60-cycle flicker) of processing sensory stimuli. A typical symptom of hypersensitivity, avoiding wearing clothes, may stem from aberrant temporal processing of texture (Green and Ben-Sasson 2010). Another study described the superior ability of individuals with ASD to temporally process visual stimuli (Falter et al. 2012). In that study, vertical bars were consecutively presented to the left and right of a fixation cross on a monitor with different temporal lag times ranging from 8.3 to 99.6 ms. The participants were instructed to determine whether the stimuli were presented simultaneously or not. The authors found that individuals with ASD judged the stimuli to not be simultaneously presented more frequently than controls, indicating that they may have superior temporal resolution. On the other hand, individuals with ASD have also been shown to have lower temporal resolution while processing tactile stimulation (Tommerdahl et al. 2008; Wada et al. 2014). Tommerdahl et al. (2008) reported lower temporal resolution in the tactile temporal order judgment (TOJ) of the index and middle fingers of one hand in individuals with ASD, although the tactile TOJ of both hands was not significantly different between the ASD and TD groups. Moreover, the temporal resolution of one hand came to be precise compared with TD individuals when conditioning vibrotactile stimuli (frequency, 25 Hz) were presented on another skin site. In contrast, Wada et al. (2014) reported that while the temporal resolution of tactile TOJ for both hands was slightly lower in children with ASD than in TD children, the p-value for this comparison was not so significant, given the large individual differences in sensory processing in the ASD group.

The contrasting results for both sensitivity and temporal resolution of sensory processing in individuals with ASD indicate a diversity in sensory processing in this population. The type of sensory processing underlying hyper-reactivity remains unclear and seems to be related to the temporal processing of stimuli from the environment. In this study, we elucidated the relationship between individual differences in temporal resolution of sensory processing and those in the severity of hypersensitivity. We focused on the tactile modality, given the variety of findings related to tactile temporal processing (Puts et al. 2014; Tommerdahl et al. 2008; Wada et al. 2014). We adopted the TOJ task with vibrotactile stimuli to measure the temporal resolution of stimulus processing and compare it between the ASD and TD groups.

## Methods

### Participants

#### Temporal Order Judgement Task

Twelve individuals with a clinical diagnosis of ASD were recruited from parent groups for children with developmental disorders and the Hospital of National Rehabilitation Center for Persons with Disabilities. An occupational therapist (M.S.) confirmed the diagnosis using the Autism Diagnostic Observation Schedule, Second Edition (ADOS-2) (Lord et al. 2012). Fourteen participants were recruited to the typically developing (TD) group. We asked the participants to complete the Japanese version of the Autism Spectrum Quotient (AQ) scale (Baron-Cohen et al. 2001; Wakabayashi et al. 2004). As one who was initially recruited as the TD adults had very high AQ (37; cut-off, 33) and ADOS-2 reciprocal social interaction subscale (6; cut-off, 4) scores, this participant can be regarded as AS condition and was included in the ASD group. (Wheelwright et al. 2010) with the final number of participants in each of the groups being 13 (ASD group: 12 clinically diagnosed participants + 1 participant with high autistic traits = 13; TD group, 14 initial TD participants—one participant with high autistic traits = 13). The participants’ Intelligence Quotients (IQs) were also assessed using the Wechsler Adult Intelligence Scale-Third Edition (WAIS-III). We also used the Wechsler Intelligence Scale for Children-Fourth Edition (WISC-IV) to evaluate one 15-year-old male participant (verbal comprehension = 88, perceptual reasoning = 132, working memory = 120, processing speed = 127, full-scale IQ (FSIQ) = 120). All the participants from both the groups had an FSIQ above 80 (within 2 standard deviations [SDs] of the standardized average). There were no significant differences in the age, verbal IQ, and performance IQ of the two groups (verbal IQ: *t* (23) = − 1.89, *p* = 0.07, Cohen’s *d* = 0.75, performance IQ: *t* (23) = − 1.09, *p* = 0.29, Cohen’s *d* = 0.43); the FSIQ (*t* (23) = 2.07, *p* = 0.02, Cohen’s *d* = 0.95) of the two groups was significantly different. The participant information is described in Table 1.

**Table 1:** Participant information

	Temporal order judgment task		Detection task	
	ASD group	TD group	ASD group	TD group
N (male:female)	13 (11:2)	13 (9:4)	11 (10:1)	12 (9:3)
Age, in years (range)	19.1 (14–27)	21.2 (16–31)	19.6 (17–27)	21.4 (18–32)
VIQ (range)	106.8 (76–134)	120.7 (91–147)	109.6 (85–127)	119.7 (102–147)
PIQ (range)	101.5 (87–120)	107.5 (82–129)	102.9 (87–120)	109.3 (102–147)
FSIQ (range)	103.5 (85–127)	118.3 (95–134)	106.3 (85–127)	116.3 (103–134)

*VIQ* verbal intelligence quotient, *PIQ* performance IQ, *FSIQ* full-scale IQ

#### Detection Task

Eleven individuals with a clinical diagnosis of ASD were recruited for this experiment. The same TD individuals participated in the detection and TOJ experiments. The FSIQ (WAIS-III) of participants in both groups was above 80. There were no significant differences in age, verbal IQ, performance IQ, and FSIQ between the two groups (verbal IQ: *t* (21) = − 1.87, *p* = 0.08, Cohen’s *d* = 0.78, performance IQ: *t* (21) = − 1.44, *p* = 0.17, Cohen’s *d* = 0.6, FSIQ: *t* (21) = − 1.2, *p* = 0.24, Cohen’s *d* = 0.51). All participants and their parents gave written informed consent after the study procedures had been fully explained.

### Procedure

We administered the two behavioural tasks on different days; the TOJ task was administered on day 1, and the detection task on day 2. The temporal resolution and detection threshold and sensitivity to vibrotactile stimuli were estimated from the responses in the TOJ and detection tasks, respectively. In addition, the degree of atypical sensory processing was assessed using a self-report questionnaire (Adolescent/Adult Sensory Profile, or AASP).

#### Temporal Order Judgement Task

**Fig. 1:**
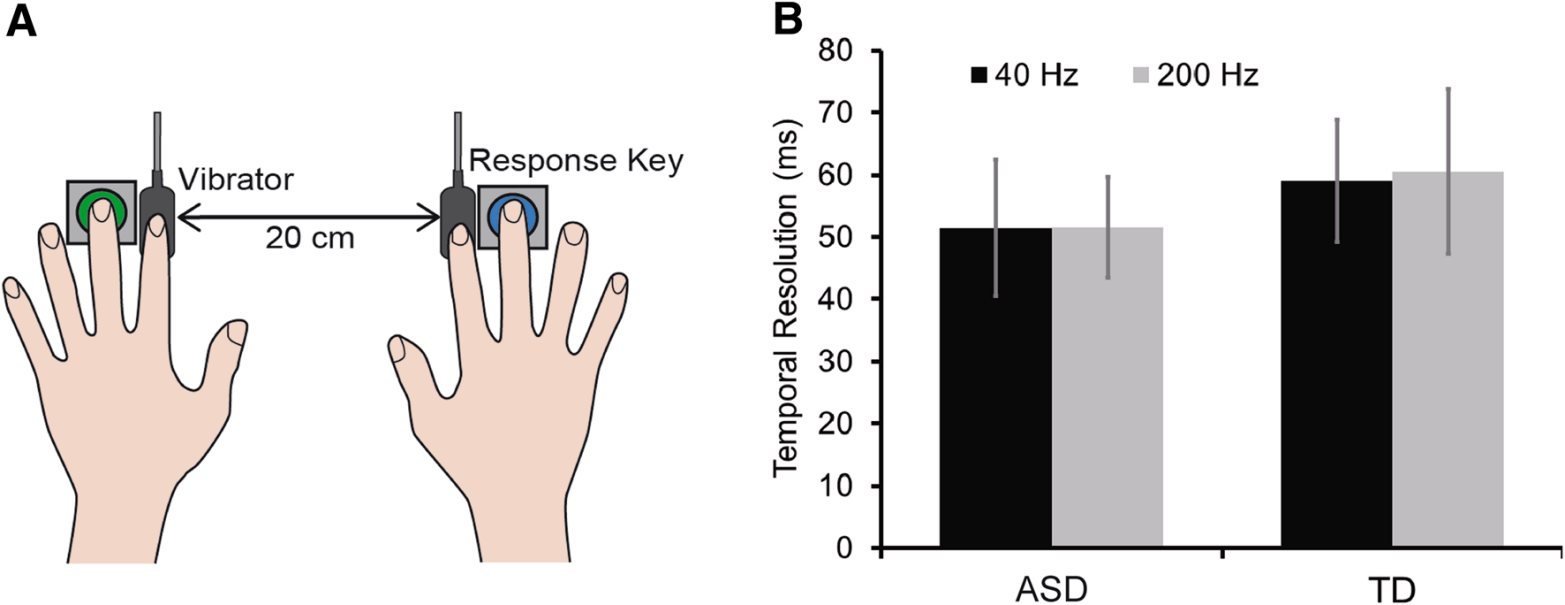
**a** Schematic representation of the TOJ task. Vibrotactile stimulation was delivered to both index fingers with a range of SOAs. The participants determined the order of the stimuli and responded by pressing a key with their middle fingers. **b** The temporal resolutions in the ASD and TD groups for the 40- and 200-Hz conditions. The error bars denote standard errors of the means

Solenoid skin contactors (FR-2007-2α, Uchida Denshi, Tokyo, Japan) were used to deliver vibrotactile stimulation (Fig. 1a). We used two frequencies of vibration (40 and 200 Hz) because Blakemore et al. (2006) reported that different responses in individuals with ASD show lower detection threshold in 200 Hz vibrotactile stimuli. It is possible that there are different response traits depending on the type of mechanoreceptors (the Pacinian and Meissner corpuscles respond to 40 and 200 Hz stimuli, respectively). The displacement (2 µm) and duration (50 ms) of the vibrations were measured by the laser displacement meter (LK-G15, KEYENCE, Osaka, Japan). White noise was presented through headphones (HD380PRO, SENNHEISER, Wedemark, Germany).

We successively delivered brief vibrotactile stimuli to the ventral surface of the participant’s left and right index fingers placed 20 cm apart, with stimulus onset asynchronies (SOAs) ranging from − 240 to 240 ms (± 15, 30, 60, 120, 240 ms), each repeated 12 times. Positive values indicate that the vibrotactile stimulus was delivered on the right index finger. Thus, each block consisted of 120 trials in each of the 40- and 200-Hz conditions (240 trials in total). The inter-stimulus intervals were randomly selected to be between 1.5 and 2.5 s. The participants were asked to determine the side to which the second stimulus was presented and respond by pressing a key as soon as possible. When the reaction time was larger than 5000 ms or the participants responded before the second stimulus, the response was excluded from the data and an additional trial was inserted at the end of the condition.

#### Detection Task

The Piezo skin contactor (FPZT-2015-1, Uchida Denshi, Tokyo, Japan) was used to deliver vibrotactile stimulation of two frequencies (40 and 200 Hz) (Fig. 3a), with stimulus displacements of 0, 1, 3, 6, 9, 12, 15, 18, 21, 24, 27, or 30 µm and duration of 500 ms, as measured by a laser displacement meter. White noise was presented through headphones.

We delivered vibrotactile stimuli to the ventral surface of the participant’s left index finger with the extent of displacement varying as described above. Each stimulus displacement condition was repeated 12 times. Thus, each block consisted of 144 trials in each of the 40- and 200-Hz conditions (288 trials in total). The participants were instructed to determine whether the stimulus was presented or not and respond by pressing a key as soon as possible after the presentation of a beep sound (pure tone, 500 Hz). The subsequent stimulus was not delivered until the subject pressed the key.

#### Subjective Ratings of Hypersensitivity

We used the AASP to evaluate the degree of responsiveness to stimuli of various modalities in their daily life (Brown et al. 2001). This self-report questionnaire consists of four subscales: low registration, sensation seeking, sensory sensitivity, and sensory avoiding. The former two categories (i.e. low registration and sensation seeking) reflect “lower responsiveness” to sensory stimuli, while the latter (i.e. sensory sensitivity and sensory avoiding) correspond to the opposite (“enhanced responsiveness”). There were no significant between-group differences in the subscales (low registration: *t* (24) = 1.55, *p* < 0.13, Cohen’s *d* = 0.61; sensation seeking: *t* (24) = − 0.18, *p* < 0.86, Cohen’s *d* = 0.07; sensory sensitivity: *t* (24) = − 0.43, *p* < 0.67, Cohen’s *d* = 0.17; sensory avoiding: *t* (24) = 0.32, *p* < 0.75, Cohen’s *d* = 0.13) or in the total sensory responsiveness (sensory sensitivity + sensation avoiding) (Cascio et al. 2008) (*t* (24) = − 0.07, *p* < 0.94, Cohen’s *d* = 0.03). Thus, we focused on the relationship between individual performances in behavioural tasks and their AASP scores.

### Data Analysis

We calculated the temporal resolution, detection threshold, and sensitivity by fitting the response data in each task to a Gaussian cumulative density function (Yamamoto and Kitazawa 2001).

In the TOJ task, the response data were sorted by the SOAs to calculate the order-judgment probability that the right index finger was stimulated later (or the left index finger was stimulated first). The judgment probabilities of the data in the TOJ task were fitted using the following function:

$$p(t)=(\mathop p\nolimits_{{\hbox{max} }} - {p_{\hbox{min} }})\int_{{ - \infty }}^{t} {\mathop {\frac{1}{{\sqrt {2\pi } \mathop \sigma \nolimits_{t} }}\exp \left( {\frac{{\mathop { - (\tau - dt)}\nolimits^{2} }}{{2\mathop \sigma \nolimits_{t}^{2} }}} \right)}\nolimits^{} } dt\tau +{p_{\hbox{min} }}$$

where *t, d*_*t*_, *σ*_*t*_, *P*_*max*_, and *P*_*min*_ represent the SOAs, size of the horizontal transition, temporal resolution, and upper and lower asymptotes of the judgment probability, respectively. The *σ*_*t*_ corresponded to the stimulation interval that yielded 84% correct responses (relative to the asymptote). We used the MATLAB optimization toolbox (MathWorks, Natick, MA, USA) for fitting to minimize the Pearson’s Chi square statistic, which reflects the discrepancy between the sampled order-judgment probability and the prediction using the four-parameter model. SPSS statistics 23 (IBM Corp., Armonk, NY, USA) was used to analyse the statistical significance of the data.

In the detection task, the data were sorted by stimulus displacements to calculate the stimulus detection probabilities. The probabilities in the detection task were fitted by the following function corresponding to the TOJ task:

$$p(t)=({p_{\hbox{max} }} - {p_{\hbox{min} }})\int_{{ - \infty }}^{t} {\mathop {\frac{1}{{\sqrt {2\pi } \mathop \sigma \nolimits_{d} }}\exp \left( {\frac{{\mathop { - (\tau - dd)}\nolimits^{2} }}{{2\mathop \sigma \nolimits_{d}^{2} }}} \right)}\nolimits^{} } dd\tau +{p_{\hbox{min} }}$$

where *t, d*_*d*_, *σ*_*d*_, *P*_*max*_, and *P*_*min*_ represent the extent of displacement of vibration, size of the horizontal transition, sensitivity, and upper and lower asymptotes of the detection probability. The *d*_*d*_ and *σ*_*d*_ values corresponded to the extent of stimulus displacement and the steepness of the function, respectively, that yielded 50% correct responses. Thus, in the detection task, we defined *d*_*d*_ as the detection threshold and *σ*_*d*_ as the detection sensitivity.

## Results

### Temporal Resolution of Processing Vibrotactile Stimuli

We examined whether the temporal resolution (*σ*_*t*_) of processing vibrotactile stimuli was different between the ASD and TD groups or not (Fig. 1b). We found no significant difference between the groups (*F* (1, 24) = − 0.32, *p* = 0.57, partial *η*^2^ = 0.013). A previous study have also demonstrated that the temporal resolution in individuals with ASD is comparable with that in TD individuals (Puts et al. 2014) in agreement with the current result. In addition, there was no significant between-group difference in frequencies (*F* (1, 24) = − 0.04, *p* = 0.85, partial *η*^2^ = 0.002) or any group × frequency interaction (*F* (1, 24) = − 0.03, *p* = 0.87, partial *η*^2^ = 0.001).

**Fig. 2:**
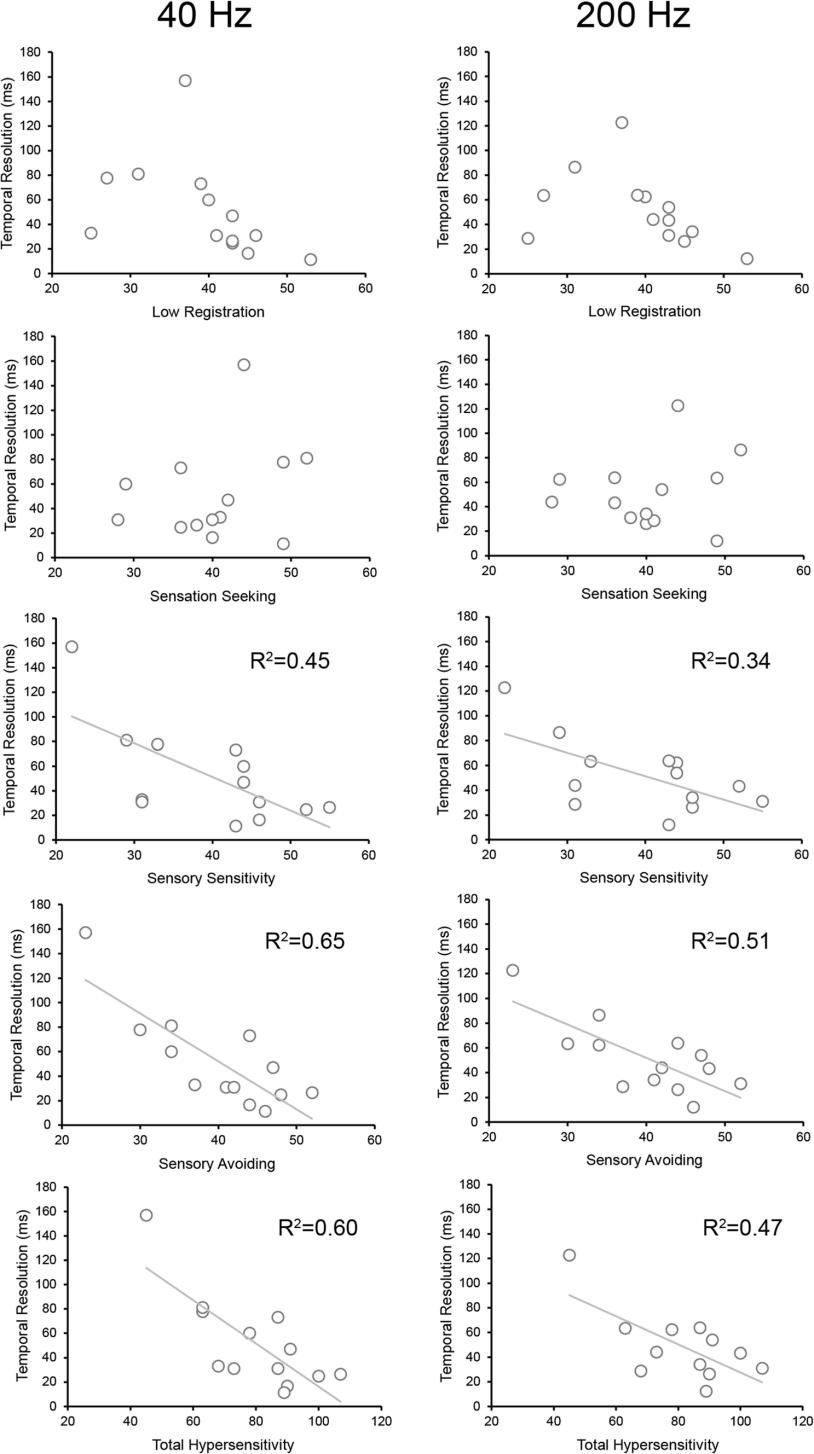
Relationship of the temporal resolution of vibrotactile stimulus processing and degree of responsiveness to various stimuli with the AASP subscales in the ASD group for the 40- and 200-Hz conditions. Solid lines indicate significant correlations

Next, we examined whether the individual differences in temporal resolution and those in sensory hypersensitivity were related to each other. There was a significant correlation (Pearson’s rank correlation coefficient) between the extent of temporal resolution (40 and 200 Hz) and the subjective ratings (AASP) for the “enhanced responsiveness” and “total sensory responsiveness” subscales [*sensory sensitivity*—40 Hz: *r* = − 0.68, *p* = 0.01, power (1 − *β*) = 0.97; 200 Hz: *r* = − 0.63, *p* = 0.02, power (1 − *β*) = 0.95; *sensory avoiding*—40 Hz: *r* = − 0.81, *p* = 0.0006, power (1 − *β*) = 0.997; 200 Hz: *r* = − 0.75, *p* = 0.003, power (1 − *β*) = 0.99); *total sensory sensitivity*—40 Hz: *r* = − 0.72, *p* = 0.005, power (1 − *β*) = 0.98; 200 Hz: *r* = − 0.72, *p* = 0.005, power (1 − *β*) = 0.98] in the ASD group (Fig. 2). In contrast, there was no relationship between the temporal resolution and the subjective ratings in the “lower responsiveness” subscale (*low registration*—40 Hz: *r* = − 0.45, *p* = 0.12, power (1 − *β*) = 0.67; 200 Hz: *r* = − 0.43, *p* = 1.57, power (1 − *β*) = 0.73; *sensory exploring*—40 Hz: *r* = 0.24, *p* = 0.41, power (1 − *β*) = 0.43; 200 Hz: *r* = 0.17, *p* = 0.58, power (1 − *β*) = 0.089). We did not find any significant correlation between the temporal resolution and the subjective ratings for any of the AASP categories in the TD group (Supplementary Table 1). There was no correlation between the temporal resolution and the ADOS-2 total and subscale scores in the ASD group (Supplementary Table 2).

### Detection Threshold and Sensitivity in Vibrotactile Stimulus Processing

**Fig. 3:**
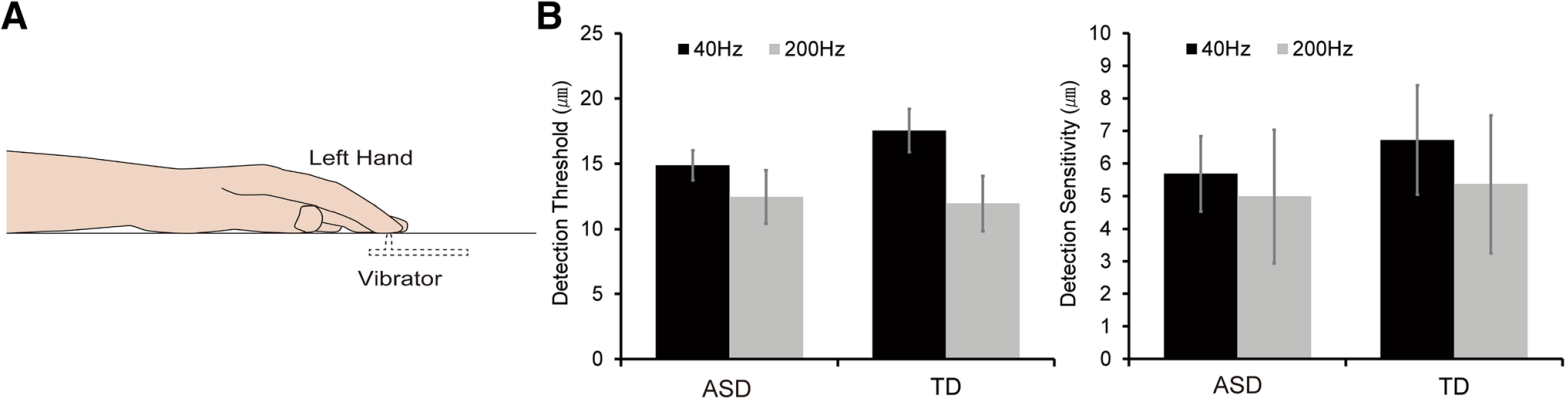
**a** Schematic representation of the detection task. Tactile stimulation was delivered to the left index finger with a range of stimulus displacements. The participants determined whether they felt the vibrotactile stimuli and responded by pressing a key after a beep sound was presented. b The detection threshold (left) and sensitivity (right) in ASD and TD groups for the 40- and 200-Hz conditions. The error bars denote standard errors of the means

We compared the detection thresholds (*d*_*d*_) for vibrotactile stimuli between the stimulus conditions (40 Hz and 200 Hz) and between the ASD and TD groups for each condition (Fig. 3b). We found a significant difference between the 40-Hz and 200-Hz conditions (*F* (1, 21) = 6.34, *p* = 0.02, partial *η*^2^ = 0.23), which may have been caused by higher sensitivity of Pacinian corpuscles than that of Meissner corpuscles (Bolanowski et al. 1988; Mountcastle et al. 1972; Talbot et al. 1968). There was no significant difference between the groups (*F* (1, 21) = 0.282, *p* = 0.6, partial *η*^2^ = 0.13) or a significant group × frequency interaction (*F* (1, 21) = 0.99, *p* = 0.33, partial *η*^2^ = 0.045). Moreover, we found no significant difference in the detection sensitivity between the groups (*F* (1, 21) = 0.62, *p* = 0.44, partial *η*^2^ = 0.015) or frequencies (*F* (1, 21) = 0.19, *p* = 0.67, partial *η*^2^ = 0.009) or a significant group × frequency interaction (*F* (1, 21) = 0.17, *p* = 0.69, partial *η*^2^ = 1.42).

In contrast to the results of the TOJ task, there were no significant correlations (Pearson’s rank correlation coefficient) between the detection threshold/sensitivity (40 and 200 Hz) and the AASP subjective ratings in the ASD and TD groups (Supplementary Tables 3 and 4), except for a slight significant correlation in sensory avoiding in the ASD group (*r* = − 0.61, *p* = 0.04, power (1 − *β*) = 0.56).

**Fig. 4:**
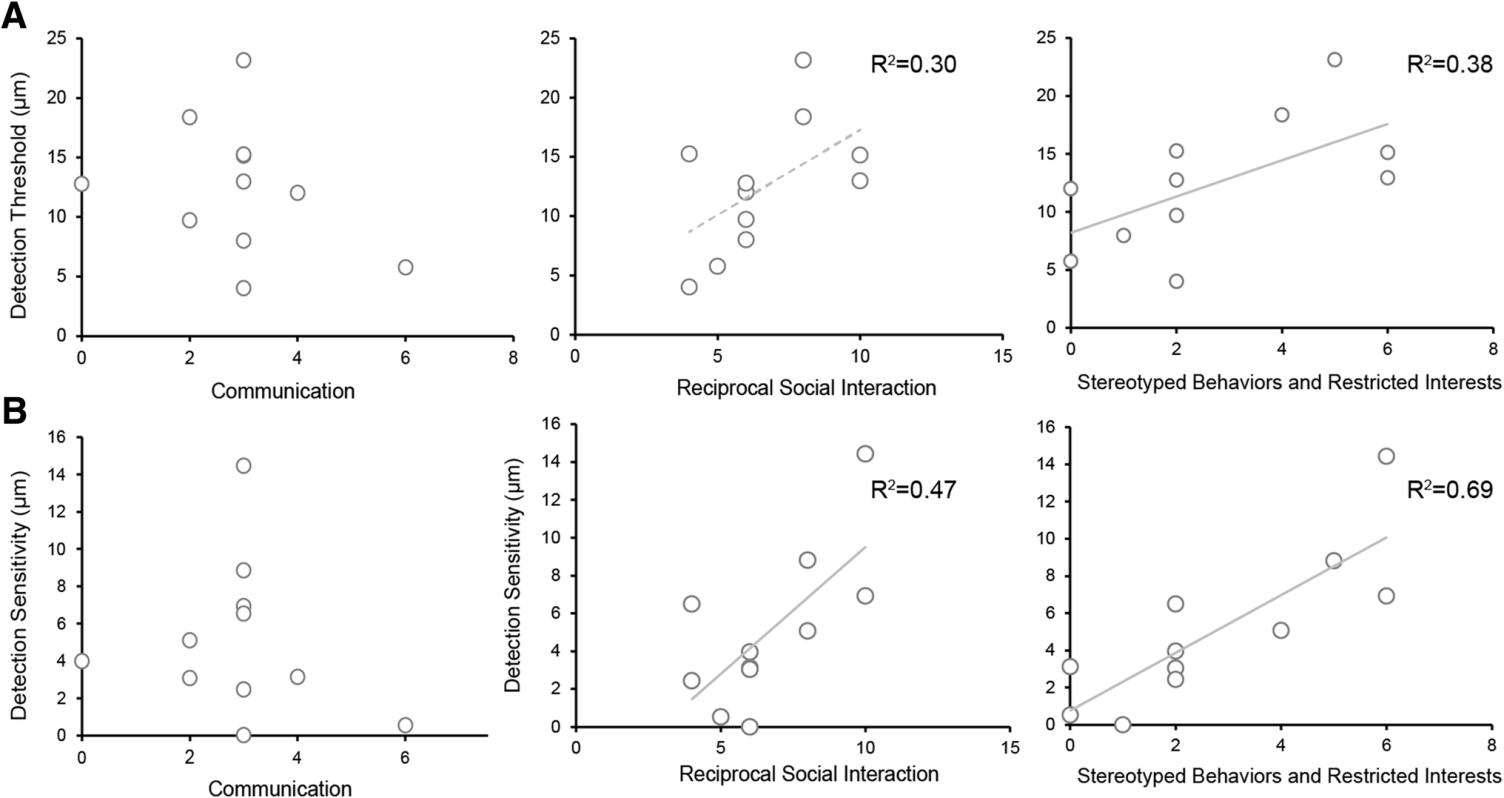
Relationship of the detection threshold (**a**) and detection sensitivity (**b**) with the severity of atypical behaviour as assessed by the Autism Diagnostic Observation Schedule, Second Edition (ADOS-2) in the ASD group for the 200-Hz condition. Solid lines indicate significant correlations and the dotted line indicates marginally significant correlation

Instead, we found that the detection threshold was correlated with the stereotyped behaviour and restricted interests’ subscale of the ADOS-2 (*r* = 0.66, *p* = 0.04, power (1 − *β*) = 0.66) and marginally correlated with the reciprocal social interaction subscale (*r* = 0.54, *p* = 0.08, power (1 − *β*) = 0.43) in the ASD group only in the 200-Hz condition (Fig. 4a) (Supplementary Tables 5 and 6). Furthermore, we found that the detection sensitivity was positively correlated with the reciprocal social interactions (*r* = 0.64, *p* = 0.017, power (1 − *β*) = 0.71) and stereotyped behaviours and restricted interests (*r* = 0.82, *p* = 0.001, power (1 − *β*) = 0.94) subscales in the ASD group only in the 200-Hz condition (Fig. 4b). There were no significant correlations observed in the 40-Hz condition.

## Discussion

Previous studies have demonstrated large individual differences in sensory processing, particularly in detection sensitivity and temporal resolution of processing vibrotactile stimuli, among individuals with ASD. As temporal processing is often considered to be linked to sensory hypersensitivity, we elucidated whether individual differences in temporal resolution are related to the degree of hyper-reactivity in patients with ASD. Our results suggest that individuals in the ASD group who had higher temporal resolution of processing vibrotactile stimuli tended to be more affected by various sensory stimuli experienced as a part of their daily life. However, the detection threshold and sensitivity were almost not related to this atypical responsiveness but were related to the severity of stereotyped behaviour and restricted interests and, partially, to reciprocal social interactions as assessed by the ADOS-2. These data indicate that temporal processing of tactile stimuli may underlie sensory hyper-reactivity in individuals with ASD, while the detection threshold and sensitivity may underlie the severity of other aspects of ASD.

Our data provide first evidence that temporal processing of stimuli, and not detection threshold and sensitivity, is correlated with a self-assessed score of hypersensitivity. In fact, the temporal resolution and detection threshold/sensitivity were not significantly different between the two groups. This kind of behavioural performance points to the existence of a wide continuum in the degree of hyper-reactivity. Wada et al. (2014) reported lower temporal resolution in children with ASD; however, the mean age of their sample (mean age, 11.8 years) was lower than that of our sample (mean age, 19.1 years). Dysfunctions according to developmental changes in sensory processing might contribute to the differences attributed to the participant groups. Since the temporal resolution of stimuli in neurotypical individuals reportedly increases from childhood to adolescence (Stevenson et al. 2017), it is possible that deviations in temporal resolution appear in individuals with ASD until adolescence. Interestingly, we found relationships between temporal resolution and hypersensitivity related to the ‘enhanced responsiveness’ and ‘total sensory responsiveness’ AASP sub-categories (and not the ‘lower responsiveness’ sub-category). These results suggest that the temporal processing of tactile stimuli is predominantly associated with hyper-reactivity when some types of sensory information included in the AASP such as visual, auditory, somatosensory, olfactory, and gustatory are provided in daily life.

Furthermore, we found that the detection threshold and sensitivity to 200-Hz vibrotactile stimuli were positively correlated with the reciprocal social interactions and stereotyped behaviour and restricted interests ADOS-2 sub-scores. Guclu et al. (2007) also indicated that elevated tactile sensitivity was related to socioemotional problems experienced in daily life. Moreover, the detection threshold was lower in individuals with Asperger’s syndrome than in their TD peers, predominantly for high frequency vibrotactile stimuli (Blakemore et al. 2006), although this result could not be replicated in another ASD sample (Guclu et al. 2007). Thus, while temporal processing of vibrotactile stimuli is linked to the degree of hypersensitivity, detection threshold/sensitivity for high frequency vibrotactile stimuli might be linked to stereotyped behaviour and restricted interests and other social impairments characteristic of ASD. However, as our sample size was small, further studies are required to examine this link between the severities of several aspects of ASD symptoms and detection performances while processing high frequency vibrotactile stimuli.

With regard to the neural basis of atypical responses to sensory stimuli, mainly hyper-reactivity, idiosyncratic somatosensory evoked potentials for tactile stimuli have been reported (Miyazaki et al. 2007). Cascio et al. (2015) also reported that early (120–220 ms) and late (220–270 ms) brain waves elicited by air-puff stimulation might be related to the degrees of hyper-reactivity and hypo-reactivity, respectively. Hyper-reactivity would then be consistent with the somatosensory association cortical response, while hypo-reactivity would be consistent with later brain processes such as allocation of attention or ascribing emotional valence to stimuli. Simon et al. (2017) demonstrated that the degree of hypo-reactivity was associated with elevated levels of left alpha and theta power and increased alpha and theta connectivity in resting state electroencephalography in toddlers at high risk (HR) of being diagnosed with ASD in the future. They also found that hypo-reactivity was related to reduced signal complexity at occipital and temporal electrodes. These findings indicate that reduced sensory responsiveness in HR toddlers corresponds to broad changes in neural synchronization, both within and across cortical areas, and a resultant loss of complex neural interactions.

Several studies using mouse models of autism have reported that autistic mice frequently show an excitation-inhibition imbalance (i.e. E/I imbalance) in the central nervous system (Braat and Kooy 2015; Pizzarelli and Cherubini 2011; Rubenstein and Merzenich 2003). One of the major features of the model mice is reduced concentration of gamma-aminobutyric acid (GABA) in the brain that involves the deactivated GABA receptor and subsequent degraded release of the neurotransmitter. The model mice showed defensive behaviour to air-puff stimulation at the whisker more frequently than wild-type mice (He et al. 2017), in addition to more frequent pathognomonic behaviour while interacting with cage mates and deficits in social communication. Similarly, human post-mortem studies showed reduced concentrations of GABA in the anterior cingulate cortex and fusiform gyrus in patients with ASD (Oblak et al. 2010). Recent magnetic resonance spectroscopy studies showing in vivo GABA states in the human brain have revealed the relationship between GABA concentrations in the human brain and behavioural performances. Robertson et al. (2016) demonstrated that GABA concentration in the visual cortex was lower in individuals with ASD than in their TD peers. Moreover, individuals with ASD also showed decreased suppressive ratio of perceptual switching when different visual stimuli were concurrently presented to each eye (i.e. binocular rivalry). Puts et al. (2015) reported reduction in GABA levels also in individuals with Tourette syndrome, with lower GABA concentrations in the somatosensory cortex being associated with more severe tics. Another study (Terhune et al. 2014) showed that reduced GABA levels might lead to more precise estimates of the temporal duration of visual stimuli. It is possible that the reduction in GABA levels in the primary sensory cortices underlies perceptual states in various sensory modalities, resulting in the inhibition of sensory inputs and involuntary movement, and sometimes in excessive sensory processing. We speculated that the enhanced temporal resolution in individuals with ASD might be caused by an E/I imbalance in their brains, and future studies are needed to further elucidate this hypothesis.

Aberrant sensory processing in patients with ASD is regarded as the basis of their impairments in social cognition and adaptive behaviour (Ben-Sasson et al. 2009). Green et al. (2018) showed that task-irrelevant tactile stimulation complicates the comprehension of the meaning of “sarcasm”, which is needed to interpret communicative intents in non-literal language. In the task, neural activities in the left auditory language areas (angular gyrus) and the occipital cortex degraded by the distractive tactile stimuli with strong activation in the somatosensory cortex. The degraded neural activity would reflect that they shifted their attention away from the task and towards the sensory stimuli. In contrast, the degradation in neural responses disappeared when they were required to shift their attention to the facial expression and tone of voice of the speaker, while the medial prefrontal cortex (mPFC) strongly activated. Thus, strong mPFC activation was assumed to inhibit distractive sensory inputs in order to properly interpret the communicative intents. These findings including our present results indicate that several stages exist regarding the occurrence factor of hyper-reactivity. In one stage, weak inhibitory function on sensory inputs and resulting strong neural activation in the primary sensory cortex would be an important factor. Higher temporal resolution of sensory stimuli may be related with this stage because this feature of sensory processing would result in vast amounts of inflow of sensory information. Difficulty in attentional deprivation from distractive and/or unpleasant feelings of sensory stimuli may play an important role at another stage. Aversive touch was found to facilitate neural activation in the posterior cingulate cortex and the insula in individuals with ASD (Cascio et al. 2012), and the amplitude of the activity in the insula was positively correlated with the severity of disabilities in social communication measured by the ADOS-2. Thus, we speculate that excessively strong neural responses by sensory inputs in connection with weak inhibitory function exist at the first stage, and then, this over-responsivity would interfere with adaptive social communication and emotional processing.

Our study provides the first report indicating that the temporal processing of vibrotactile stimuli may underlie sensory hypersensitivity, while detection threshold/sensitivity for high frequency vibrotactile stimuli may be linked to the severity of some ASD symptoms. Enhanced sensory processing in patients with ASD may result in a large inflow of sensory signals from the surrounding environment, the several neural substrates contributing to the diversity of sensory processing in these patients. Thus, treatment plans must consider individual sensory sensitivity levels, which may consequently determine the patients’ compliance to treatment.

## Electronic supplementary material

Below is the link to the electronic supplementary material.

Supplementary material 1 (DOCX 60 KB)
