# Supplementary material for: Higher Tactile Temporal Resolution as a Basis of Hypersensitivity in Individuals with Autism Spectrum Disorder
Source: J Autism Dev Disord. 2018 Jul 17;49(1):44–53. doi: 10.1007/s10803-018-3677-8 (PMC6331495; doi:10.1007/s10803-018-3677-8)
Supplement: Supplementary file 1 — Supplementary material 1 (DOCX 60 KB) [file 10803_2018_3677_MOESM1_ESM.docx]

**Journal of Autism Developmental Disorders:**

**Higher tactile temporal resolution as a basis of hypersensitivity in individuals**

**with autism spectrum disorder**

Masakazu Ide^1.2*^, Ayako Yaguchi^1, 3^, Misako Sano^4, 5^, Reiko Fukatsu^4, 6^, Makoto Wada^1^

^1^Developmental Disorders Section, Department of Rehabilitation for Brain Functions, Research Institute of National Rehabilitation Center for Persons with Disabilities, Saitama, Japan

^2^Japan Society for the Promotion of Science, Tokyo, Japan

^3^Department of Contemporary Psychology, Rikkyo University, Saitama, Japan

^4^Information and Support Center for Persons with Developmental Disorders, National Rehabilitation Center for Persons with Disabilities, Saitama, Japan

^5^National Rehabilitation Center for Children with Disabilities, Tokyo, Japan

^6^Department of Rehabilitation for Brain Functions, Research Institute of National Rehabilitation Center for Persons with Disabilities, Saitama, Japan

*Corresponding author: Masakazu Ide

Developmental Disorders Section, Department of Rehabilitation for Brain Functions Research Institute of National Rehabilitation Center for Persons with Disabilities

4-1, Namiki, Tokorozawa-shi, Saitama, 359-8555, Japan

Phone: +81-4-2995-3100

E-mail: ide-masakazu@rehab.go.jp

**Supplementary Table 1**. Relationship of the temporal resolution of vibrotactile stimulus processing and degree of responsiveness to various stimuli with the AASP sub-scales in the ASD and TD groups for the 40 Hz and 200 Hz conditions.

|  |  | Row Registration | Sensory Seeking | Sensory Sensitivity | Sensory Avoiding | Total Hypersensitivity |
| --- | --- | --- | --- | --- | --- | --- |
| ASD | 40 Hz | R = -0.45  P = 0.12  Power (1-β) ­= 0.35 | R = 0.25  P = 0.41  Power (1-β) ­= 0.13 | R = -0.68  P = 0.01^*^  Power (1-β) ­= 0.77 | R = -0.81  P = 0.00006***  Power (1-β) ­= 0.96 | R = -0.77  P = 0.002**  Power (1-β) ­= 0.92 |
|  | 200 Hz | R = -0.43  P = 0.14  Power (1-β) ­= 0.32 | R = 0.17  P = 0.02  Power (1-β) ­= 0.09 | R = -0.63  P = 0.02^*^  Power (1-β) ­= 0.69 | R = -0.75  P = 0.003**  Power (1-β) ­= 0.9 | R = -0.72  P = 0.005**  Power (1-β) ­= 0.85 |
| TD | 40 Hz | R = 0.35  P = 0.24  Power (1-β) ­= 0.22 | R = -0.25  P = 0.41  Power (1-β) ­= 0.13 | R = 0.2  P = 0.52  Power (1-β) ­= 0.1 | R = -0.005  P = 0.99  Power (1-β) ­= 0.05 | R = 0.28  P = 0.34  Power (1-β) ­= 0.16 |
|  | 200 Hz | R = 0.28  P = 0.36  Power (1-β) ­= 0.15 | R = 0.48  P = 0.1  Power (1-β) ­= 0.39 | R = 0.35  P = 0.23  Power (1-β) ­= 0.22 | R = -0.23  P = 0.45  Power (1-β) ­= 0.12 | R = -0.06  P = 0.85  Power (1-β) ­= 0.05 |

**Supplementary Table 2**. Relationship of the temporal resolution of vibrotactile stimulus processing and the severity of atypical behaviour as assessed by the Autism Diagnostic Observation Schedule, Second Edition (ADOS-2) in the ASD group for the 40 Hz and 200 Hz conditions.

|  | Communication | Reciprocal Social Interaction | Stereotyped Behaviors and Restricted Interests |
| --- | --- | --- | --- |
| 40 Hz | R = -0.25  P = 0.4  Power (1-β) ­= 0.13 | R = 0.25  P = 0.44  Power (1-β) ­= 0.13 | R = 0.35  P = 0.24  Power (1-β) ­= 0.22 |
| 200 Hz | R = -0.31  P = 0.3  Power (1-β) ­= 0.18 | R = 0.15  P = 0.64  Power (1-β) ­= 0.08 | R = 0.22  P = 0.47  Power (1-β) ­= 0.11 |

**Supplementary Table 3**. Relationship of the detection threshold of vibrotactile stimulus processing with the AASP sub-scales in the ASD and TD groups for the 40 Hz and 200 Hz conditions.

|  |  | Row Registration | Sensory Seeking | Sensory Sensitivity | Sensory Avoiding | Total Hypersensitivity |
| --- | --- | --- | --- | --- | --- | --- |
| ASD | 40 Hz | R = -0.47  P = 0.14  Power (1-β) ­= 0.32 | R = 0.25  P = 0.46  Power (1-β) ­= 0.11 | R = -0.36  P = 0.27  Power (1-β) ­= 0.19 | R = -0.61  P = 0.04^*^  Power (1-β) ­= 0.56 | R = -0.44  P = 0.18  Power (1-β) ­= 0.28 |
|  | 200 Hz | R = -0.18  P = 0.14  Power (1-β) ­= 0.08 | R = 0.37  P = 0.29  Power (1-β) ­= 0.02 | R = -0.31  P = 0.38  Power (1-β) ­= 0.15 | R = -0.42  P = 0.22  Power (1-β) ­= 0.26 | R = -0.49  P = 0.14  Power (1-β) ­= 0.36 |
| TD | 40 Hz | R = -0.003  P = 0.99  Power (1-β) ­= 0.05 | R = 0.59  P = 0.05  Power (1-β) ­= 0.52 | R = 0.08  P = 0.82  Power (1-β) ­= 0.06 | R = -0.03  P = 0.92  Power (1-β) ­= 0.05 | R = 0.02  P = 0.96  Power (1-β) ­= 0.05 |
|  | 200 Hz | R = -0.06  P = 0.71  Power (1-β) ­= 0.05 | R = 0.48  P = 0.12  Power (1-β) ­= 0.35 | R = -0.06  P = 0.85  Power (1-β) ­= 0.05 | R = -0.06  P = 0.87  Power (1-β) ­= 0.05 | R = -0.07  P = 0.85  Power (1-β) ­= 0.05 |

**Supplementary Table 4**. Relationship of the detection sensitivity of vibrotactile stimulus processing with the AASP sub-scales in the ASD and TD groups for the 40 Hz and 200 Hz conditions.

|  |  | Row Registration | Sensory Seeking | Sensory Sensitivity | Sensory Avoiding | Total Hypersensitivity |
| --- | --- | --- | --- | --- | --- | --- |
| ASD | 40 Hz | R = 0.3  P = 0.37  Power (1-β) ­= 0.15 | R = -0.27  P = 0.43  Power (1-β) ­= 0.12 | R = -0.09  P = 0.79  Power (1-β) ­= 0.06 | R = 0.29  P = 0.38  Power (1-β) ­= 0.14 | R = 0.2  P = 0.56  Power (1-β) ­= 0.09 |
|  | 200 Hz | R = -0.04  P = 0.91  Power (1-β) ­= 0.05 | R = 0.003  P = 0.99  Power (1-β) ­= 0.05 | R = -0.52  P = 0.12  Power (1-β) ­= 0.39 | R = -0.4  P = 0.25  Power (1-β) ­= 0.24 | R = -0.49  P = 0.15  Power (1-β) ­= 0.35 |
| TD | 40 Hz | R = 0.3  P = 0.37  Power (1-β) ­= 0.15 | R = -0.27  P = 0.43  Power (1-β) ­= 0.12 | R = 0.09  P = 0.79  Power (1-β) ­= 0.06 | R = 0.29  P = 0.38  Power (1-β) ­= 0.14 | R = 0.2  P = 0.56  Power (1-β) ­= 0.09 |
|  | 200 Hz | R = 0.28  P = 0.13  Power (1-β) ­= 0.71 | R = 0.29  P = 0.38  Power (1-β) ­= 0.14 | R = -0.09  P = 0.8  Power (1-β) ­= 0.06 | R = -0.2  P = 0.56  Power (1-β) ­= 0.09 | R = 0.08  P = 0.81  Power (1-β) ­= 0.06 |

**Supplementary Table 5**. Relationship of the detection threshold of vibrotactile stimulus processing and the severity of atypical behaviour as assessed by the Autism Diagnostic Observation Schedule, Second Edition (ADOS-2) in the ASD group for the 40 Hz and 200 Hz conditions.

|  | Communication | Reciprocal Social Interaction | Stereotyped Behaviors and Restricted Interests |
| --- | --- | --- | --- |
| 40 Hz | R = -0.25  P = 0.4  Power (1-β) ­= 0.12 | R = 0.45  P = 0.44  Power (1-β) ­= 0.11 | R = -0.35  P = 0.24  Power (1-β) ­= 0.18 |
| 200 Hz | R = -0.31  P = 0.3  Power (1-β) ­= 0.15 | R = 0.15  P = 0.64  Power (1-β) ­= 0.07 | R = 0.22  P = 0.47  Power (1-β) ­= 0.1 |

**Supplementary Table 6**. Relationship of the detection sensitivity of vibrotactile stimulus processing and the severity of atypical behaviour as assessed by the Autism Diagnostic Observation Schedule, Second Edition (ADOS-2) in the ASD group for the 40 Hz and 200 Hz conditions.

|  | Communication | Reciprocal Social Interaction | Stereotyped Behaviors and Restricted Interests |
| --- | --- | --- | --- |
| 40 Hz | R = -0.17  P = 0.62  Power (1-β) ­= 0.08 | R = 0.05  P = 0.9  Power (1-β) ­= 0.52 | R = 0.27  P = 0.42  Power (1-β) ­= 0.12 |
| 200 Hz | R = -0.17  P = 0.61  Power (1-β) ­= 0.08 | R = 0.69  P = 0.02^*^  Power (1-β) ­= 0.71 | R = 0.62  P = 0.04^*^  Power (1-β) ­= 0.57 |
